# Optimization of a secondary VOI protocol for lung imaging in a clinical CT scanner

**DOI:** 10.1002/acm2.12354

**Published:** 2018-05-21

**Authors:** Thomas C. Larsen, Vissagan Gopalakrishnan, Jianhua Yao, Catherine P. Nguyen, Marcus Y. Chen, Joel Moss, Han Wen

**Affiliations:** ^1^ National Heart, Lung and Blood Institute National Institutes of Health Bethesda MD USA; ^2^ Rush Medical College Chicago IL USA; ^3^ Department of Radiology Hatfield Clinical Center National Institutes of Health Bethesda MD USA

**Keywords:** CT VOI protocol, high resolution, lung texture, nodule, pulmonary cyst

## Abstract

We present a solution to meet an unmet clinical need of an in‐situ “close look” at a pulmonary nodule or at the margins of a pulmonary cyst revealed by a primary (screening) chest CT while the patient is still in the scanner. We first evaluated options available on current whole‐body CT scanners for high resolution screening scans, including ROI reconstruction of the primary scan data and HRCT, but found them to have insufficient SNR in lung tissue or discontinuous slice coverage. Within the capabilities of current clinical CT systems, we opted for the solution of a secondary, volume‐of‐interest (VOI) protocol where the radiation dose is focused into a short‐beam axial scan at the z position of interest, combined with a small‐FOV reconstruction at the xy position of interest. The objective of this work was to design a VOI protocol that is optimized for targeted lung imaging in a clinical whole‐body CT system. Using a chest phantom containing a lung‐mimicking foam insert with a simulated cyst, we identified the appropriate scan mode and optimized both the scan and recon parameters. The VOI protocol yielded 3.2 times the texture amplitude‐to‐noise ratio in the lung‐mimicking foam when compared to the standard chest CT, and 8.4 times the texture difference between the lung mimicking and reference foams. It improved details of the wall of the simulated cyst and better resolution in a line‐pair insert. The Effective Dose of the secondary VOI protocol was 42% on average and up to 100% in the worst‐case scenario of VOI positioning relative to the standard chest CT. The optimized protocol will be used to obtain detailed CT textures of pulmonary lesions, which are biomarkers for the type and stage of lung diseases**.**

## 1 INTRODUCTION

In chest CTs of patients with lung disease it has been established that the texture in the vicinity of pulmonary cysts provides better differentiation of the lung disease,^1^, ^2^ and details of the margins and the texture of lung nodules help classify the lung disease.^3^, ^4^, ^5^ There is a need for a “close look” at a cyst or margin revealed by a screening scan, where a higher level of detail can improve the evaluation of disease clinically and computationally, thus improving diagnostic accuracy.^6^, ^7^ Furthermore, such a “close look” can be particularly useful as a way to diagnose early stages of lung disease when the suspicious features are small or subtle. For example, in interstitial lung disease, early signs of disease which manifest as interstitial lung abnormalities (ILA) on chest CT scans have been associated with numerous risk factors for disease.^8^, ^9^, ^10^, ^11^, ^12^, ^13^, ^14^, ^15^ A recent clinical study also concluded that changes in CT texture measures are significantly correlated with changes of pulmonary function in patients with idiopathic pulmonary fibrosis, and can be used to predict diminished function in these patients.^16^ Therefore, a detailed view of such features would allow for earlier diagnoses, faster determination and better monitoring of treatment.

In the context of quantitative CT analysis, tissue texture is the pattern of density fluctuations in small groups of pixels.^8^, ^9^, ^16^, ^17^, ^18^ The porous structure of lung tissue means a highly heterogeneous density distribution at the microscopic scale, below the resolution of clinical CT scanners. However, statistical sampling effects result in observable density fluctuation of the lung parenchyma at CT resolutions. This effect was studied by G. Kemerink and coauthors in the 1990s as a factor in the measurement of lung density.^19^, ^20^ They showed that both the texture amplitude and the image noise level increase with decreasing voxel size (increasing resolution).

There are standard options available on clinical whole‐body CT scanners to obtain high resolution in a screening chest CT. They include high‐resolution CT (HRCT) scan from the data acquisition side, as well as high‐resolution ROI reconstruction of raw data from a standard chest CT. The HRCT scan provides high resolution in 1–2 mm slices separated by 10 mm or wider gaps, and therefore can miss large portions of a cyst or miss small features entirely. The second option of ROI recon of primary scan data raises resolution but also increases the noise level. We found that the increased noise overwhelmed the texture signal in the lung‐mimicking foam.

Therefore, our solution for a “close look” at a pulmonary cyst or nodule was to perform an additional, secondary scan that achieves high resolution and low noise in a small volume identified from the primary scan. To maintain SNR at high resolution, it is necessary to raise the photon flux through the targeted volume by concentrating the radiation dose. Volume‐of‐interest CT^21^, ^22^ is an active research topic with innovative approaches in all aspects from system architecture to dynamic beam‐shaping filters to image reconstruction, some of which are summarized by review articles in the field.^23^, ^24^, ^25^, ^26^, ^27^ Some examples of experimental demonstrations include small‐animal studies^28^, ^29^ and implementations on tabletop or clinical c‐arm CBCT systems.^30^, ^31^, ^32^, ^33^, ^34^, ^35^, ^36^

However, within the capabilities of current whole‐body CT scanners, how to target a specific volume was the question we tried to answer. We chose a volume‐of‐interest protocol which combines a short‐length axial scan of low rotation speed (high mAs) at the z position of interest with a small reconstructed FOV centered at the xy location of interest. The short z length means a short beam by the collimator which reduces the Dose‐Length Product (DLP) and the Effective Dose (ED). In a chest phantom with a foam insert that had the same level of signal fluctuation as lung tissue^19^, ^20^ and contained a simulated cyst, we comprehensively evaluated the dose, scan parameters and recon voxel sizes to optimize the scan for lung disease patients, as well as to understand the interplay between these parameters.

The optimization was based on the ratio between noise‐subtracted signal fluctuation of the lung‐mimicking foam and noise in an air void. Although the foam does not simulate the microscopic structures of lung parenchyma, its pixel‐wise signal fluctuation nonetheless comes from microscopic density distributions. Such a signal fluctuation encompasses all underlying textures, and is therefore a measure of the ability of a CT scan to detect such textures.

## 2 MATERIALS AND METHODS

### 2.A Lung tissue‐mimicking phantom

**Figure 1:**
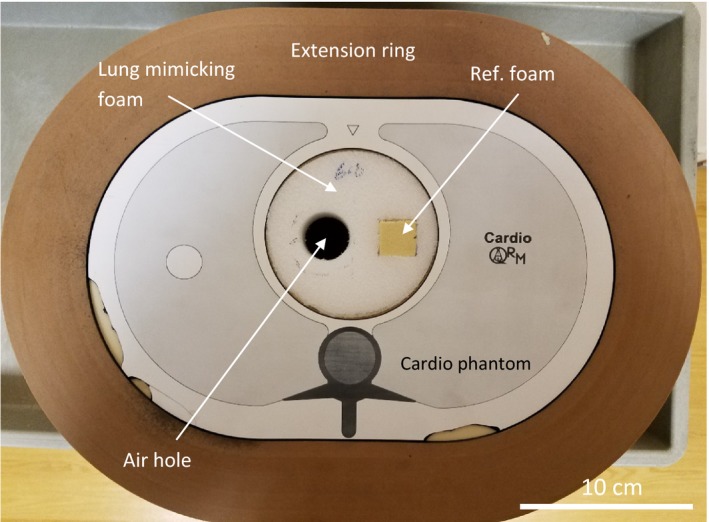
The semianthropomorphic chest phantom with a 5 cm thick extension ring to simulate a surrounding adipose layer. A foam insert is made with 96 kg/m3 polyethene foam, which mimics the texture (CT density fluctuation) of lung parenchyma. A circular air hole in the foam is lined with vasoline to simulate a cyst with a hyperintense wall. The air hole is also used to estimate image noise levels. A square section of the foam is replaced with a second less porous foam of the same average mass density as a reference material of low texture. In a 2nd part of the study, the foam insert was replaced with a standard line‐pair insert to evaluate image resolution.

To predict performance of the VOI protocols in lung imaging, we built a foam insert for a standard semianthropomorphic phantom (Cardio QRM) (Fig. 1). The phantom had a central space for inserts. Polyethylene foam with a density of 96 kg/m^3^ has been shown to have the same level of pixel‐wise signal fluctuation due to microscopic porosity as the level in normal human lung parenchyma in CT scans.^19^ The foam was cut into a cylinder to fit as an insert. Two holes were cut into the foam. One hole was left vacant to create an air hole for image noise measurements. The second hole was filled with a reference polyurethane foam of the same density as the lung‐mimicking foam but minimal signal fluctuation due to its uniform composition, as a control sample. The air hole was also lined with vasoline to simulate a walled structure similar to the cystic lesions in Lymphangioleiomyomatosis (LAM) patients.^37^ The chest phantom was placed in a standard extension ring of 5 cm thickness to simulate adipose tissue around the body.

The insert space of the standard phantom was at its center, while its lung cavities had a uniform filling material and were inaccessible. Therefore, our foam insert was at the center of the phantom instead of the lung cavities. Besides the practical reason, both noise and resolution are generally dependent on the absolute position in the FOV as well as proximity to dense structures that give out artifacts. Given the complexity of this issue, our study was a comparative study in the same central location that is less influenced by these factors.

In a separate visual evaluation of the image resolution, the foam insert was replaced with the standard line‐pair insert for the chest phantom.

### 2.B CT scan and image reconstruction protocols

**Figure 2:**
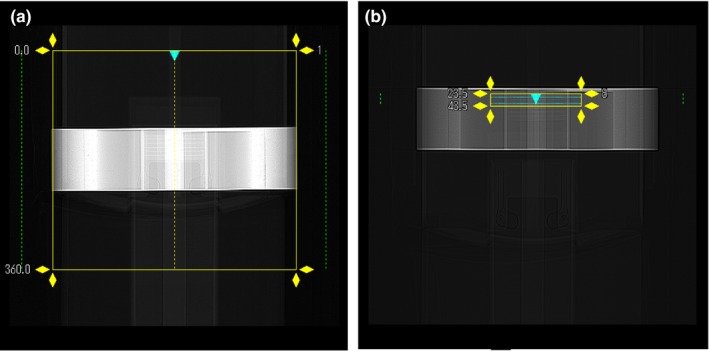
Screen shots from the whole‐body CT scanner showing the prescribed scan volume in (a) the standard helical chest scan protocol and (b) an axial scan of a VOI protocol. Coronal scout radiographs of the chest phantom are shown, with the scanner axis shown as vertical. Axial z positions are labeled in mm. The default z length of the standard scan is 360 mm. The z length of all VOI protocols is 20 mm

The study was performed on a Toshiba Aquillion One Genesis CT system. The standard clinical chest CT protocol was a helical scan with tube settings of 120 kV/R700 mA, rotation speed of 0.275 s per turn, a total scan time of 3.6 s to cover 360 mm length of the chest, FOV size of 400 mm, and helical scan pitch of 0.813[Fig. 2(a)]. The VOI protocols were single‐rotation axial (static bed) scans of 20 mm z length (minimum allowed by the scanner in axial scan mode), and reduced FOV [Fig. 2(b)]. Four different sets of axial scan parameters were tested (Table 1). These had a tube voltage of 120 kV, tube currents of 350–700 mA, and rotation speeds of 2–3 s per turn, which were also the total scan times. The prescribed FOV size only influenced the recon area, not the reported dose, which will be described below. The focal spot size of the scanner was small (nominal 0.8/0.9 mm) up to 350 mA tube current and large (nominal 1.5/1.6 mm) at 700 mA, hence the 350 mA current was chosen as one of the tested settings. The common point of all VOI protocols was an axial scan of minimum z length which provided high photon fluxes through a small volume. All VOI protocols copied the exact same z location, which was first positioned manually in the chest phantom.

**Table 1:** The complete list of the noise levels for all combinations of scan settings and recon settings that were studied. The columns are different scan settings and the rows are different recon settings. The scan settings are denoted with a prefix “A” if they are the VOI protocol axial scan, or “H” for the standard helical chest scan, followed by the scanner settings of tube current(mA)/gantry rotation time per turn (s), then CTDIvol(mGy)/dose‐length product(mGy*cm). The recon settings are labeled with (field of view), (in‐plane pixel size)x(slice thickness), all in mm units

Recon parameters	Scan protocol				
	H700 mA/0.275s #bib8.3 mGy/342.6 mGy*cm	A350 mA/2s, 47.5 mGy/95.1 mGy*	A350 mA/3 s, 71 mGy/142 mGy*	A700 mA/2s, 101.5 mGy/203 mGy*	A700 mA/3 s, 151.6 mGy/303.2 mGy*
400, 0.78 × 1, #	16.5 ± 0.7*	10.0 ± 0.3	8.3 ± 0.3	7.5 ± 0.4	6.5 ± 0.3
400, 0.78 × 0.5, #	19.2 ± 0.9	11.7 ± 0.6	10.0 ± 0.4	8.8 ± 0.4	7.6 ± 0.3
329, 0.64 × 0.5, #	28.6 ± 0.9	17.3 ± 0.7*	14.2 ± 0.4	12.6 ± 0.5	10.3 ± 0.2
300, 0.59 × 0.5, #	34.8 ± 0.9	19.9 ± 0.9	17.3 ± 1.2*	14.2 ± 0.6	11.8 ± 0.2
240, 0.47 × 0.5, #	43.4 ± 1.4	23.3 ± 1.2	18.7 ± 0.3	16.2 ± 0.6*	13.6 ± 0.3
212, 0.41 × 0.5, #	47.5 ± 1.1	24.0 ± 1.1	19.4 ± 0.4	17.2 ± 1.3	13.8 ± 0.3
150, 0.29 × 1	48.0 ± 1.5	22.7 ± 1.1	18.4 ± 0.6	15.7 ± 0.7	13.4 ± 0.5
150, 0.29 × 0.5, #	57.5 ± 2.0	27.7 ± 1.7	21.8 ± 0.5	18.4 ± 0.7	16.6 ± 1.3*

The “#” marks recon settings of relatively cubic voxels with ratios of (pixel size/slice thickness) between 0.58 and 1.56. The “*” denotes combinations having noise levels equivalent to the standard chest scan with the standard recon setting for lung imaging.

For dose evaluation, dose values reported by the scanner, including CTDIvol and DLP are listed in Table 1. The Effective Dose (ED), required by our IRB for research CT protocols, is defined as the biological effects from a nonuniform, partial‐body exposure in terms of a whole‐body exposure. Current guidelines include organ specific sensitivity in the form of the ICRP 103 organ weighting factor.^38^ For the average ED estimate in a patient population where the scan location can be anywhere in the lungs, the average DLP to ED conversion factor of a chest scan that covers all organs in the chest was used.^38^ For the worst‐case ED, our medical physicist modeled a scan placed on the most sensitive organ in the chest, which was the breast, using the ImPACT spreadsheet and the ICRP 103 organ weighting factor, with input of the specific scanner model parameters by the CT vendor.

The image recon matrix size of the scanner was 512 × 512 in plane, with user‐adjustable recon FOV and slice thickness. The published effective detector pixel size at isocenter is 0.5 mm. Following the manufacturer's recommendation for chest CT, the recon kernel FC52 was used for both standard and VOI protocols. A range of FOV/pixel sizes and slice thicknesses were tested for each protocol. Table 1 shows all combinations of scan and recon settings that were evaluated for noise levels. Recon settings with relatively cubic voxels, where (pixel size/slice thickness) ratio was between 0.58 and 1.56, were evaluated for foam texture measurements as potential candidates for patient application. More elongated voxels were excluded for texture analysis to avoid extra averaging effect in the z direction, which reduces the texture amplitude.

### 2.C Noise, foam texture measurements and resolution evaluation

Separate scans were performed in the chest phantom for the foam insert and the standard line‐pair insert. All images were analyzed with the software Osirix (http://www.osirix-viewer.com). Noise and foam texture measurements were made with the foam insert data. Statistical comparisons were made with unpaired t‐tests. Visual comparisons of image resolution were made with the line‐pair insert data.

**Figure 3:**
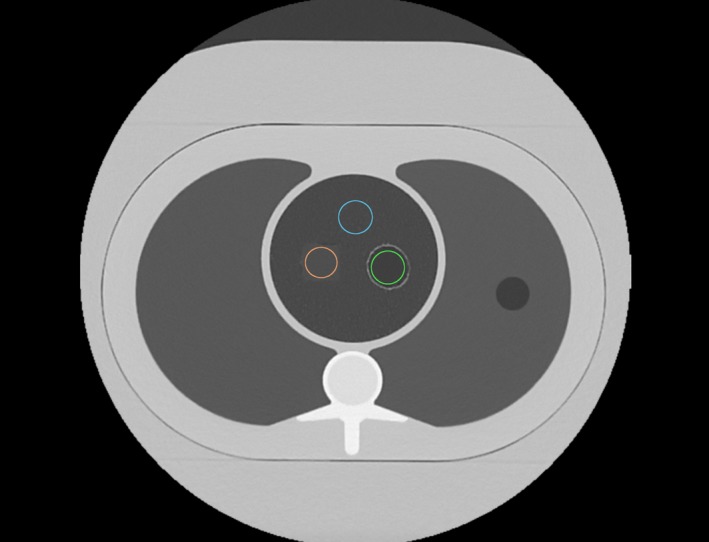
A cross‐section image of the chest phantom with the foam insert, showing the regions used for the noise measurement in the air hole (green circle), signal fluctuation (texture amplitude) measurements in the lung‐mimicking foam (blue circle) and in the control foam (pink circle). The image is one of the 10 z locations over the 20 mm z length of the VOI protocol where the measurements were made.

Referring to Fig. 3, image noise measurements were made in the air hole in the foam insert as the standard deviation of the CT value. Foam texture amplitude measurements were made in both the lung‐mimicking foam and in the control foam. All measurements were made on a per slice basis in 10 slices that were evenly distributed over the 20 mm z length of the VOI protocol. The same set of 10 z locations were used for all measurements for consistency. The average and standard deviation of the 10 measurements for each quantity were used for statistics.

For each scan setting, the noise levels in the air hole *σ*_*A*_ for different recon slice thickness and FOV were plotted as a function of 1/*V* ^0.5^, where *V* is the reconstructed voxel volume.^19^ By the noise measurements, the recon settings for the VOI protocols that yielded the same noise level as the standard scan and recon setting were found.

We adopted Kemerink et al.'s measure of the visibility of the foam textures as the texture amplitude‐to‐noise ratio,^19^ *A*_*T*_/*σ*_*A*_, on a per slice basis. The texture amplitude *A*_*T*_ of the foams, defined as the noise‐subtracted amplitude of the texture signal, were quantified according to the equation

$$A_{T}=\sqrt{\sigma_{F}^{2}-\sigma_{A}^{2}}$$

where *σ*_*F*_ is the standard deviation of the CT values in the foams.

To evaluate whether the VOI protocol improves the differentiation of materials of different textures, we looked at a spectrum of 26 texture properties and calculated the differences between the lung mimicking foam and the reference foam. These texture properties have been shown to have varying degrees of clinical relevance in the literature.^1^, ^2^, ^17^, ^18^

In the resolution study with the line‐pair insert in the chest phantom, the smallest visible lines were visually determined. Visual comparisons were made in two ways. One way was to compare scan/recon combinations that gave equivalent noise levels. The other was to compare the standard scan with the optimal VOI protocol at the same recon setting. In the latter, the optimal VOI protocol was chosen as the scan/recon combination that yielded the highest texture amplitude‐to‐noise ratio in the lung mimicking foam.

Additionally, noise power spectra of the standard and the optimal VOI protocol were measured in the air hole over the 10 z locations. These were compared with each other to support the difference in line pair resolution between the two protocols.

## 3 RESULTS

**Figure 4:**
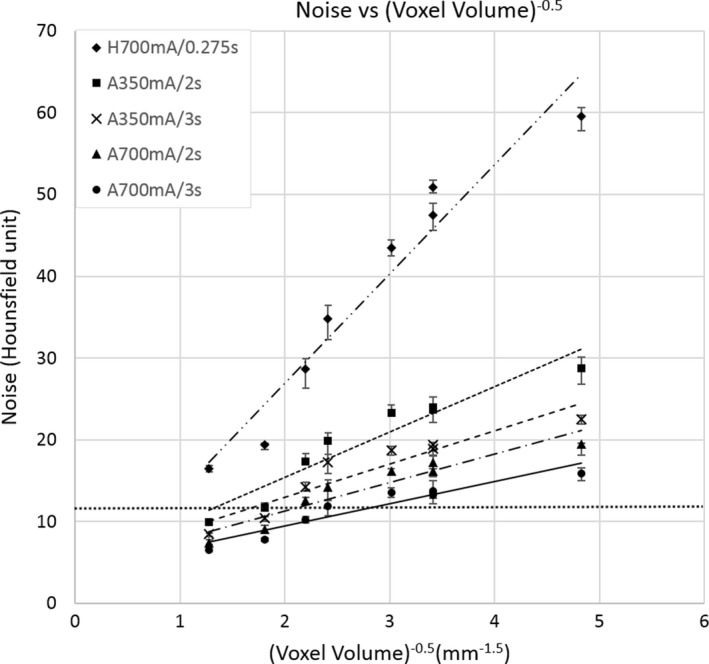
Noise vs. 1/(recon voxel volume)^0.5^ for all combinations of scan and reconstruction settings listed in Table 1. Noise is the standard deviation of CT values in the air hole of the foam insert. The scan settings are denoted with a prefix “A” if they are the VOI protocol axial scan, or “H” for the standard helical chest scan, followed by the tube current(mA)/gantry rotation time per turn (s). Linear regression lines for each scan protocol are shown. The horizontal dotted line marks the noise level of the standard scan and recon setting for chest CT on our scanner. VOI combinations of the same noise level have smaller recon voxel volumes. Error bars are standard deviations (n = 10).

The measured random noise level in the air hole of the lung‐mimicking foam for all scan/recon combinations are listed in Table 1. The relationship of noise level versus 1/(recon voxel volume)^0.5^ is plotted in Fig. 4. The relationship is approximately linear for each scan protocol.^19^ For a given recon setting, the VOI protocols had higher CTDIvol and correspondingly lower noise levels than the standard scan (Table 1). For example, at the 150 mm FOV/0.5 mm slice thickness recon setting, the ratio of noise between the standard scan and the 350 mAs/3s VOI protocol was 2.64 (*P* = 2.4E‐14), and the corresponding inverse ratio of CTDIvol was 8.55.

**Figure 5:**
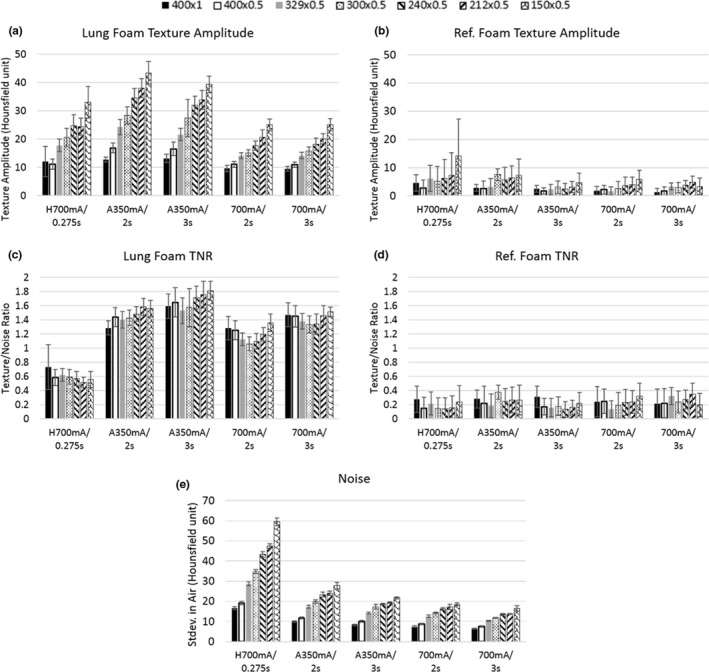
Measured foam texture visibility and image noise levels for all scan settings and recon voxels of relatively cubic dimensions (marked “#’ in Table 1). Data bars are grouped by scan settings which are labeled by tube current(mA)/rotation time per turn(s) in the horizontal axes with prefix “H” for the standard chest scan and “A” for the axial scans. Recon settings are represented by different hash patterns and denoted as FOV(mm) × slice thickness(mm) in the top legend. (a), (b) are noise‐subtracted texture amplitudes of the lung mimicking and reference foams respectively. The foams have the same mass density but different porosity. (c), (d) are the texture amplitude‐to‐noise ratio for the two types of foam. (e) is the measured image noise level, which is the standard deviation of the CT values in the air hole in the foam insert. Error bars are standard deviations (*n* = 10).

Figure 5 summarizes the foam texture visibility *A*_*T*_/*σ*_*A*_ defined in Section 2.C for all scan protocols and recon settings of relatively cubic voxels (marked by “#” in Table 1). In the lung‐mimicking phantom, the VOI protocol of 350 mA/3s yielded the highest texture amplitude‐to‐noise ratio (TNR) with the recon setting of 150 mm FOV/0.5 mm slice. Specifically, the TNR increased from 0.56 of the standard scan to 1.81 of the 350 mA/3s VOI protocol (*P* = 6.6E‐14). The TNR of the 350 mA/3s VOI protocol was higher than the shorter 350 mA/2s VOI protocol (1.81 vs. 1.56, *P* = 1.7E‐4). It was also higher than the 700 mA/3s VOI protocol having a large focal spot size (1.81 vs. 1.51, p = 1.4E‐5). The optimal VOI protocol was taken as the scan and recon settings that yielded the highest TNR in the lung‐mimicking foam, which was 350 mA/3s with recon of 150 mm FOV and 0.5 mm slice, voxel size of 0.29 × 0.29 × 0.5 mm^3^.

**Figure 6:**
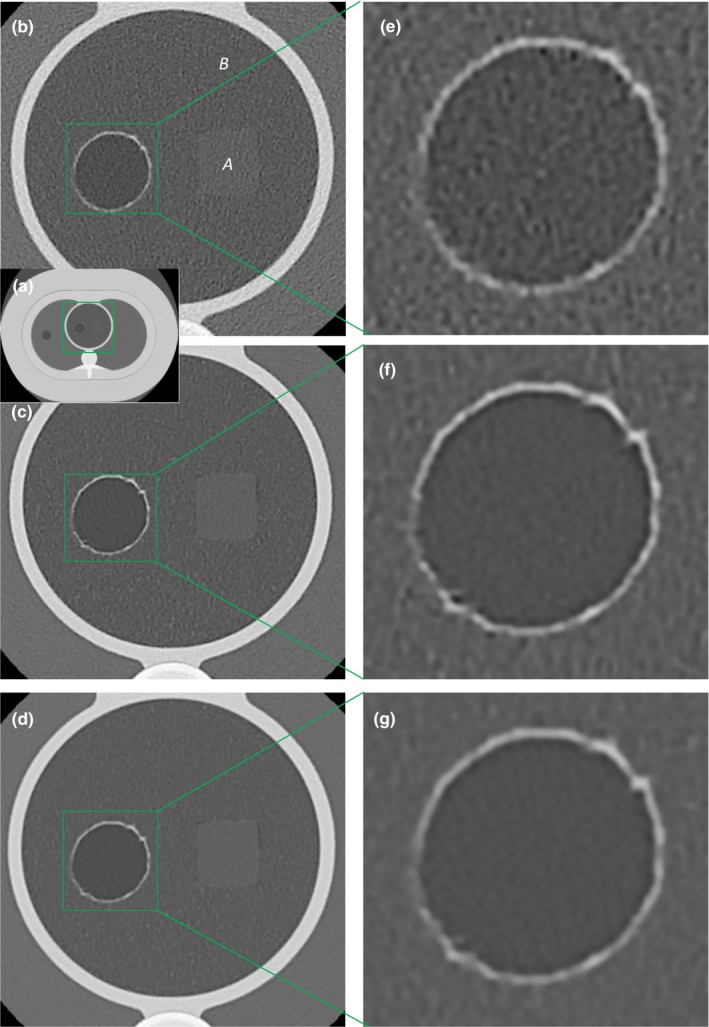
Visual comparisons in the lung‐mimicking foam insert in the chest phantom among the standard 700 mA/0.275s helical scan, the 350 mA/3 s VOI protocol and the 700 mA/3 s VOI protocol, all reconstructed to the same voxel size of 0.29 mm in‐plane by 0.5 mm slice. Window/level settings are identical. (a) is an over‐view of the chest phantom. The foam insert is outlined by the green square. (b), (c), and (d) are magnified views of the foam insert from the standard #bib350–700 mA/3 s VOI protocols respectively. The texture difference between the reference foam “A” and the lung mimicking foam “B” is visible only in the VOI protocols. The foams have the same mass density but different chemical composition, which gave slightly different average CT values. (e), (f), and (g) are magnified views of the air hole from the three scans. Detail of the vasoline‐lined wall is more distinct in the VOI protocols. The air hole simulates a lung cyst having a hyper‐intense rim. The 700 mA/3 s scan is more blurred than the 350 mA/3 s scan, due to the switch from small to large focal spot sizes.

Visual comparison of the foams among the standard, the 350–700 mA/3s VOI protocols are shown in Fig. 6. The “grainy” noise in the air hole is reduced by the VOI protocols. At the same time, the texture difference between the lung‐mimicking and the reference foams became distinct, and the detail of the wall of the air hole became better defined. The 700 mA VOI protocol appears more blurred than the 350 mA VOI protocol due to the larger focal spot size.

Measurements of clinically relevant texture properties in the lung mimicking and the reference foams are summarized in Table 2 in terms of the difference between the two. The recon setting was 150 mm FOV/0.5 mm slice thickness. The optimal VOI protocol and the standard scan were compared. In the standard scan, 14 texture properties had significantly different values between the foams. In the VOI protocol, 16 texture properties had significantly different values between the foams. Of the texture properties that had significantly different values in both the standard and VOI protocols (13 texture properties), 12 properties had higher differences in the VOI protocol. On average, texture differences in the VOI protocol were 8.4 times those in the standard scan.

**Table 2:** Difference of texture properties between the lung‐mimicking and the reference foams. Measurements from the optimal VOI protocol and the standard scan are compared at the same recon voxel size of 0.29 × 0.29 × 0.5 mm^3^. Of the 26 texture properties evaluated, 13 had significantly different values between the two types of foam in both the standard and VOI protocols. These are listed. Of the 13, 12 properties had higher differences in the VOI protocol. On average, texture differences were 7.4 times higher in the VOI protocol compared to the standard

Texture property	Standard H700 mA/0.275s		A350 mA/3 s VOI		Ratio of VOI/standard
	Difference between two foams	*P* value	Difference between two foams	*P* value	
Run percentage	0.027	<1E‐15	0.459	<1E‐15	17
High gray run emphasis	5.33	<1E‐15	5.723	<1E‐15	1.073734
Long run low gray emphasis	0.3692	<1E‐15	0.707	<1E‐15	1.914951
Inverse difference	0.0326	<1E‐15	0.1579	<1E‐15	4.843558
Low gray run emphasis	0.1252	<1E‐15	0.22559	<1E‐15	1.801837
Short run emphasis	0.0063	<1E‐15	0.1304	<1E‐15	20.69841
Short run low gray emphasis	0.0906	<1E‐15	0.15136	<1E‐15	1.67064
Entropy	0.134	6.68E‐13	1.242	<1E‐15	9.268657
Skewness	0.2988	4.63E‐12	0.63662	<1E‐15	2.130589
Short run high gray emphasis	3.945	1.3E‐10	1.883	<1E‐15	0.477313
Mean	31.6	3.07E‐06	43.3	<1E‐15	1.370253
Long run emphasis	0.071	0.000173	2.952	<1E‐15	41.57746
Inertia	0.112	0.000231	0.591	<1E‐15	5.276786

**Figure 7:**
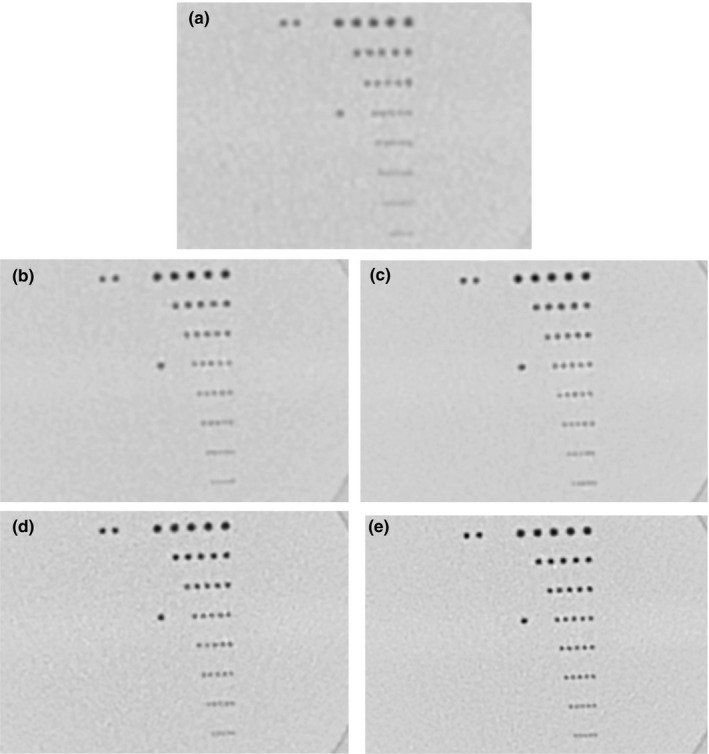
Visual comparison of image resolution by the line‐pair insert in the chest phantom. Recon settings that gave the same noise level are shown for all scan protocols, denoted by “*” in Table 1. Window/level settings are identical. (a) is the standard chest CT protocol of helical 700 mA/0.275s, recon 400 mm FOV × 1 mm slice thickness. (b) to (e) are from the VOI protocols. Denoted as scanned with tube current(mA)/scan time(s) and recon FOV(mm) × Slice thickness(mm), these are: (b) 350 mA/2s, 329 mm × 0.5 mm; (c) 350 mA/3 s, 300mmx0.5 mm; (d) 700 mA/2s, 240 mm × 0.5 mm; (e) 700 mA/3 s, 150 mm × 0.5 mm. The 7.14 lp/cm lines are resolved by the 700 mA/3 s scan. The standard scan marginally resolved 4.16 lp/cm lines.

**Figure 8:**
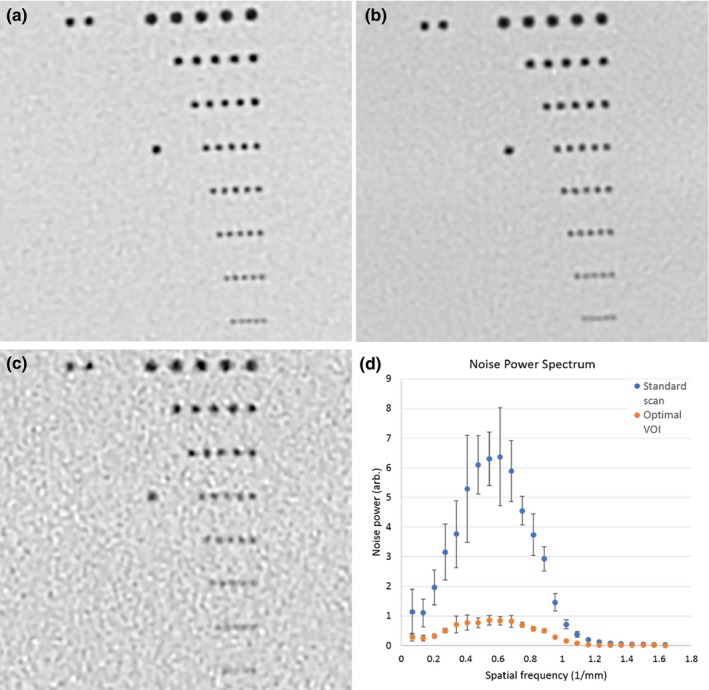
Visual comparison of image resolution among scan protocols, all reconstructed to the same setting of 150 mm FOV by 0.5 mm slice thickness, 0.29 × 0.29 × 0.5 mm3 voxel size. Window/level settings are identical. (a) The 350 mA/3 s VOI protocol resolved 8.33 lp/cm lines. (b) The 700 mA/3 s VOI protocol resolved 7.14 lp/cm lines. (c) The standard 700 mA/0.275s helical chest scan marginally resolved 6.25 lp/cm lines. (d) Noise spectra of the standard 700 mA/0.275s helical chest scan and the 350 mA/3 s VOI protocol at the same recon setting of 150 mm FOV by 0.5 mm slice thickness. Mean and standard deviation over the 10 z locations through the 20 mm z length of the VOI protocol are plotted.

Visual comparison of resolution in the line‐pair insert are illustrated in Figs. 7 and 8. The comparison was made in two ways. One was to compare scan/recon combinations that produced the same noise level in the air hole of the foam insert as the standard scan/recon combination for chest CT. These combinations are marked by “*” in Table 1. Results are summarized in Fig. 7. The VOI protocols supported smaller recon voxel volumes at the same noise level, which resulted in the visibility of denser lines. For example, the standard scan/recon setting had a voxel size of 0.8 mm in‐plane by 1 mm slice, which resolved lines of 4.16 lp/cm density; a VOI protocol of 700 mA/3s with a recon voxel size of 0.29 mm in‐plane by 0.5 mm slice yielded the same noise level, which resolved 7.14 lp/cm.

The second visual comparison of resolution was amongst standard and VOI protocols using the same recon setting of 150 mm FOV (0.29 mm pixel size) and 0.5 mm slice thickness. Results are summarized in Fig. 8. The VOI protocol of 350 mA/3s was previously determined in the foam insert to yield the highest TNR in the lung‐mimicking foam. Here, it resolved 8.33 lp/cm lines. It had better resolution than the 700 mA/3s VOI protocol, which resolved 7.14 lp/cm lines. The standard scan marginally resolved 6.25 lp/cm lines. The improvement of line‐pair resolution was due to reduced noise level, which is quantified in a comparison of the noise power spectrum between the optimal VOI protocol and the standard protocol at the same reconstruction FOV [Fig. 8(d)].

## 4 DISCUSSION

On a clinical CT scanner, we found that by concentrating the radiation into a short scan length combined with reconstruction in a small FOV, it was feasible to increase the texture visibility of a lung‐mimicking foam in a chest phantom by multiple fold through improvement of resolution and through reduction in noise. On our scanner, the optimal VOI protocol was determined to be an axial scan of 20 mm z length, 350 mA and 3 s scan, reconstructed at 150 mm FOV with voxel dimensions of 0.29 × 0.29 × 0.5 mm^3^. The texture amplitude‐to‐noise ratio of the VOI protocol was 3.2 times that of the standard chest scan, which was in agreement with the value of sqrt(1/CTDIvol ratio) = 2.92, and also consistent with the 1:2.64 ratio of the image noise level.

Assuming the scan location to be randomly distributed in the chest in a population of patients, the average effective dose of the VOI protocol was estimated at 2.6 mSv. For the worst‐case scenario where the scan was focused on the breasts, the ED reached 6.2 mSv. Since the VOI protocol follows the standard chest CT, the overall dose in the worst‐case scenario doubles the level of an exam without the VOI. Regarding the portion of the dose that is applied in the VOI protocol, a fundamental question about focusing radiation is dose–response linearity: given a fixed amount of radiation, is the risk of cancer the same whether the radiation is spread over a larger volume compared to a smaller volume? At the dose levels of clinical diagnostic imaging devices, including interventional procedures that often reach several hundred mGray (several fold the CTDIvol of our VOI protocol), the current guidelines for ED estimation is based on the consensus of linear response as the best option.^39^

A major concern of the VOI protocol for lung imaging is motion blurring: the slow gantry rotation speed makes them more susceptible to cardiac pulsatile motion during the scan time. A possible solution to the problem is a prospectively cardiac‐gated axial scan of multiple high‐speed rotations over several heart beats in a breath‐hold. Prospectively gated scans are available on some clinical scanners for cardiovascular studies. It will be investigated as an alternative to the single‐rotation axial scan in this study.

In this work, the optimization of the VOI protocol was performed on a specific scanner and we expect that the optimal parameters will vary by scanner models. Despite scanner variability, most clinical scanners support axial scans of short z length for such a VOI protocol. What we found specific to the scanner used in the study was that for the VOI protocol, the foam texture visibility was limited by the focal spot size. The evidence was that the 700 mA/3 s scan with the large focal spot size had a lower noise level but also lower TNR than the 350 mA/3 s scan of smaller focal spot size. Another finding, likely also scanner specific, was that the prescribed scan FOV had no significant effect on the dose values as reported by the scanner. It might indicate that the lateral width of the beam was not particularly adjusted with the prescribed FOV.

Besides lung imaging, the usefulness of an additional, on‐the‐spot, secondary VOI protocol with high local radiant fluence could also be considered for other situations where a high‐resolution ROI recon of the primary scan data generates too much noise for the structure of interest, for example in tumors and microcalcification. Whether the added diagnostic value warrants the incremental dose of a secondary scan is likely specific to the exam and the patient.

## CONFLICT OF INTEREST

The authors have no conflicts of interest.
